# Hypoxia-induced lncRNA-NUTF2P3-001 contributes to tumorigenesis of pancreatic cancer by derepressing the miR-3923/KRAS pathway

**DOI:** 10.18632/oncotarget.6830

**Published:** 2016-01-07

**Authors:** Xiang Li, Shi-jiang Deng, Shuai Zhu, Yan Jin, Shi-peng Cui, Jing-yuan Chen, Cheng Xiang, Qun-ying Li, Chi He, Shu-feng Zhao, Heng-yu Chen, Yi Niu, Yang Liu, Shi-chang Deng, Chun-you Wang, Gang Zhao

**Affiliations:** ^1^ Pancreatic Disease Institute, Union Hospital, Tongji Medical College, Huazhong University of Science and Technology, Wuhan, China; ^2^ Department of Medical Ultrasound, Union Hospital, Tongji Medical College, Huazhong University of Science and Technology, Wuhan, China

**Keywords:** HIF-1α, lncRNAs, miRNAs, KRAS, pancreatic cancer

## Abstract

Recent studies indicate that long non-coding RNAs (lncRNAs) play crucial roles in numerous cancers, while their function in pancreatic cancer is rarely elucidated. The present study identifies a functional lncRNA and its potential role in tumorigenesis of pancreatic cancer. Microarray co-assay for lncRNAs and mRNAs demonstrates that lncRNA-NUTF2P3-001 is remarkably overexpressed in pancreatic cancer and chronic pancreatitis tissues, which positively correlates with KRAS mRNA expression. After downregulating lncRNA-NUTF2P3-001, the proliferation and invasion of pancreatic cancer cell are significantly inhibited both *in vitro* and *vivo*, accompanying with decreased KRAS expression. The dual-luciferase reporter assay further validates that lncRNA-NUTF2P3-001 and 3′UTR of KRAS mRNA competitively bind with miR-3923. Furthermore, miR-3923 overexpression simulates the inhibiting effects of lncRNA-NUTF2P3-001-siRNA on pancreatic cancer cell, which is rescued by miR-3923 inhibitor. Specifically, the present study further reveals that lncRNA-NUTF2P3-001 is upregulated in pancreatic cancer cells under hypoxia and CoCl2 treatment, which is attributed to the binding of hypoxia-inducible factor-1α (HIF-1α) to hypoxia response elements (HREs) in the upstream of KRAS promoter. Data from pancreatic cancer patients show a positive correlation between lncRNA-NUTF2P3-001 and KRAS, which is associated with advanced tumor stage and worse prognosis. Hence, our data provide a new lncRNA-mediated regulatory mechanism for the tumor oncogene KRAS and implicate that lncRNA-NUTF2P3-001 and miR-3923 can be applied as novel predictors and therapeutic targets for pancreatic cancer.

## INTRODUCTION

The long non-coding RNAs (lncRNAs) are a class of non-coding RNAs with more than 200 nucleotides in length [1], which account for the vast majority of transcripts in human genome compared with the protein-coding RNAs [2, 3]. It has reported that lncRNAs can serve as molecular signals to regulate transcription; as decoys to titrate protein and transcription factors; as guides to recruit chromatin-modifying enzymes; and as scaffolds to bring together multiple proteins to form ribonucleoprotein complexes [4]. Accumulative evidences establish the participation of lncRNAs in human disease pathogenesis including malignant neoplasm. Research from Takayama et al. showed that overexpression of lncRNA-CTBP1-AS promoted prostate cancer cell proliferation by directly inhibiting CTBP1 expression [5]. Yang et al. revealed that lncRNA-HNF1A-AS1 was highly expressed in oesophageal adenocarcinoma and promoted cell invasion and growth by modulation of chromatin and nucleosome assembly as well as by H19 induction [6]. The other researches also demonstrated that lncRNA-HOTTIP/HOXA13 and lncRNA-HEIH participated in the modulation of liver cancer cell metastasis and progression [7, 8]. In pancreatic cancer, data demonstrates that some differentially regulated lncRNAs are correlated with prognosis in patients with pancreatic cancer [9–12]. Li et al. found that lncRNA-HOTTIP participated in pancreatic cancer progression and gemcitabine resistance via HOXA13 regulation [13]. Moreover, lncRNA-ENST00000480739 was also identified as a tumor suppresser in pancreatic ductal adenocarcinoma by regulating OS-9 and HIF-1α [14]. However, the function of dysregulated lncRNAs in pancreatic cancer is not fully elucidated. In present study, the microarray co-assay for lncRNA and mRNA discovered that lncRNA-NUTF2P3-001 was significantly increased in both pancreatic cancer and chronic pancreatitis tissues compared with noncancerous pancreatic tissues. Moreover, lncRNA-NUTF2P3-001 was positively correlated with KRAS mRNA expression. Since KRAS is widely considered as promoter in pancreatic cancer tumorigenesis [15], thus we speculate that lncRNA-NUTF2P3-001 might be a functional lncRNA in pancreatic cancer by regulating KRAS expression.

Some lncRNAs are identified as competing endogenous RNAs (ceRNAs) which act as molecular sponges for a microRNA through their miRNAs binding sites (also referred to as miRNAs response elements, MREs), thereby derepressing the targets of the respective miRNAs. LncRNA-PTENP1 has been discovered as ceRNAs to protect PTEN from miRNAs-mediated degradation [16]. Recently, lncRNA-MD1 has been reported to regulate the myoblasts differentiation was regulated by acting as sponge to protect MyoD mRNA from the inhibition of miR-133 and miR-135 [17]. Similarly, LncRNA-RoR is overexpressed in embryonic stem cells and essential for the maintenance of Core TFs by binding to miR-145 [18]. Very interestingly, the miRNAs target prediction algorithm predicts that lncRNA-NUTF2P3-001 can combine with miR-3923, and KRAS is predicted as the top one in the 87 transcripts with conserved binding sites for miR-3923. KRAS gene mutation is found at high rates in pancreatic, thyroid, colorectal, and lung carcinoma, which is widely considered essential for carcinogenesis [19–21]. Except for that research reveals that mutation of KRAS is required in the initiation and maintenance of pancreatic cancer [22], some results further imply that the KRAS overexpression is also valuable to be uncovered for tumorigenesis. Khvalevsky et al. demonstrated that downregulating KRAS expression with RNAi obviously restrained tumor growth of pancreatic cancer model [23]. Moreover, miR-96 and miR-217 were found as inhibitors in the development of pancreatic cancer by prohibiting KRAS expression [24, 25]. Hence, we further investigate whether lncRNA-NUTF2P3-001 could act as ceRNAs to upregulate KRAS expression through competitive combination with miR-3923.

Recent studies have demonstrated that the lncRNAs can be regulated under hypoxia in cancer [26, 27]. In breast cancer, hypoxia-induced lncRNA EFNA3 leads to Ephrin-A3 protein accumulation and subsequent promoted matastasis [26]. The data from Takahashi K has showed that lncRNA-RoR is responsively increased in hypoxia and further promotes survival of hepatocellular cells by regulating miR-145-HIF pathway [28]. Since the hypoxia is also a distinct characteristic of microenvironment in pancreatic cancer [29], we further consider whether this remarkable overexpression of lncRNA-NUTF2P3-001 in pancreatic cancer is induced by the hypoxia microenvironment.

In present study, the positive correlation between lncRNA-NUTF2P3-001 and KRAS mRNA was further validated in normal pancreas, chronic pancreatitis and pancreatic cancer. Moreover, gain and loss experiments of lncRNA-NUTF2P3-001 were performed to evaluate its role in the proliferation and invasion of pancreatic cancer cell in both *vitro* and *vivo*. Meanwhile, the dual-luciferase reporter assay was applied to verify the competitive binding of lncRNA-NUTF2P3-001 and KRAS mRNA with miR-3923. Furthermore, the knock-in and knock-out of miR-3923 were performed to confirm that lncRNA-NUTF2P3-001 upregulates KRAS expression by depriving the inhibition of miR-3923 on KRAS. More importantly, ChIP assay and HREs luciferase reporter assay were further adopted to identify that lncRNA-NUTF2P3-001 is transcriptionally upregulated by HIF-1α in pancreatic cancer cell.

## RESULTS

### LncRNA-NUTF2P3-001 is overexpressed in pancreatic cancer and chronic pancreatitis tissues, which positively correlates with KRAS expression and clinical outcome of pancreatic cancer patients

**Figure 1:**
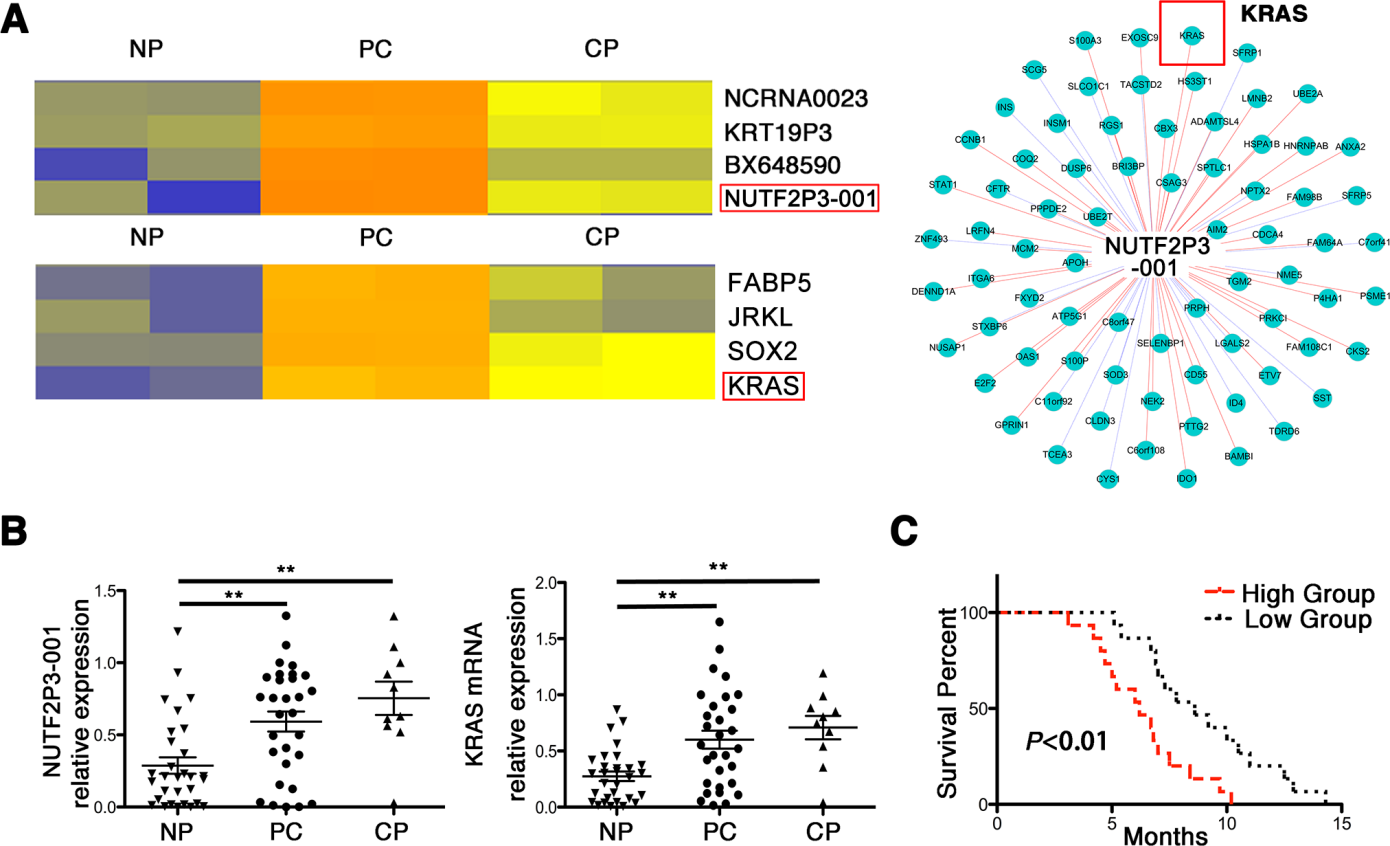
LncRNA-NUTF2P3-001 was obviously overexpressed in pancreatic cancer tissues and correlated with KRAS expression, as well as positively correlated tumor progression and worse prognosis (**A**) Hierarchical clustering analysis demonstrated the differentially expressed lncRNAs and mRNAs among noncancerous pancreatic (NP), pancreatic cancer (PC) and chronic pancreatitis (CP) tissues, including lncRNA-NUTF2P3-001-111E2.1 (NUTF2P3-001) and KRAS. The CNC (coding-non-coding) network was constructed by Markov cluster algorithm (MCL) and it showed the positive correlation of NUTF2P3-001 and KRAS mRNA expression. (**B**) The expression of NUTF2P3-001 and KRAS mRNA in samples including 30 normal pancreatic tissues (NP), 30 pancreatic cancer (PC) and 10 chronic pancreatitis (CP) was detected by qRT-PCR. (**C**) The overall survivals in 30 pancreatic cancer (PC) patients were represented by Kaplan-Meier curves. The *p*-value represents the comparison between groups (*p < 0.05, **p < 0.01).

Hierarchical clustering analysis of microarray data showed genetic alterations in the expression of both lncRNAs and mRNAs among pancreatic cancer (PC), chronic pancreatitis (CP) and noncancerous pancreatic (NP) tissues (Figure 1A left). Compared with NP tissues, lncRNA-NUTF2P3-001 (Supplementary Figure 1) was one of the significantly increased lncRNAs both in PC and CP. Followed that, data of CNC (coding-non-coding) network implied that lncRNA-NUTF2P3-001 significantly correlated with the expression of KRAS mRNA (Figure 1A right), which was also upregulated in PC and CP (Figure 1A left). The data of microarray was further validated in randomly selected tissues by qRT-PCR (30 PC, 10 CP and 30 NP tissues, Figure 1B). Furthermore, the positive correlation between lncRNA-NUTF2P3-001 and KRAS mRNA was identified according to the data of qRT-PCR in tissues, which was coincident with the CNC prediction not only in PC but also in CP specimens (Supplementary Figure S2). Afterwards, overexpressed lncRNA-NUTF2P3-001 correlated with large tumor size, poor tumor differentiation, TNM stage, lymphatic invasion, distant metastasis (Table 1) and shorter survival time of patients with pancreatic cancer (Figure 1C), which indicated that upregulated lncRNA-NUTF2P3-001 might contribute to the development of pancreatic cancer.

**Table 1:** Correlation between overexpressed lncRNA-NUTF2P3-001 and clinical characteristics of patient with pancreatic cancer

Parameters	Number of cases	NUTF2P3-001 expression		*P* Value
		High	Low	
***Gender***				
Male	20	12	8	0.121
Female	10	3	7	
***Age***				
< 60	17	8	9	0.713
≥ 60	13	7	6	
***Tumor Size (cm)***				
< 2	14	4	10	0.028*
≥ 2	16	11	5	
***Histological Grade***				
High/Moderate	20	7	13	0.020*
Low	10	8	2	
***TNM Stage***				
I–II	14	4	10	0.026*
III–IV	16	11	5	
***Lymphatic invasion***				
Positive	14	10	4	0.028*
Negative	16	5	11	
***Vascular infiltration***				
Positive	11	5	6	0.705
Negative	19	10	9	
***Distant metastasis***				
Positive	12	9	3	0.025*
Negative	18	6	12	

Note: Overexpression of NUTF2P3-001 was significantly associated with large tumor size (*P* = 0.028), poor tumor differentiation (*P* = 0.020), lymphatic invasion (*P* = 0.028), distant metastasis (*P* = 0.025) and TNM stage (*P* = 0.026), but not with patients' age (*P* = 0.713), gender (*P* = 0.121) and vascular infiltration *P* = 0.705). The *p*-value represents the comparison between groups (*p < 0.05, **p < 0.01).

### Knockdown of lncRNA-NUTF2P3-001 inhibits viability, proliferation and invasion in pancreatic cancer cell, accompanying with decreased KRAS expression

**Figure 2:**
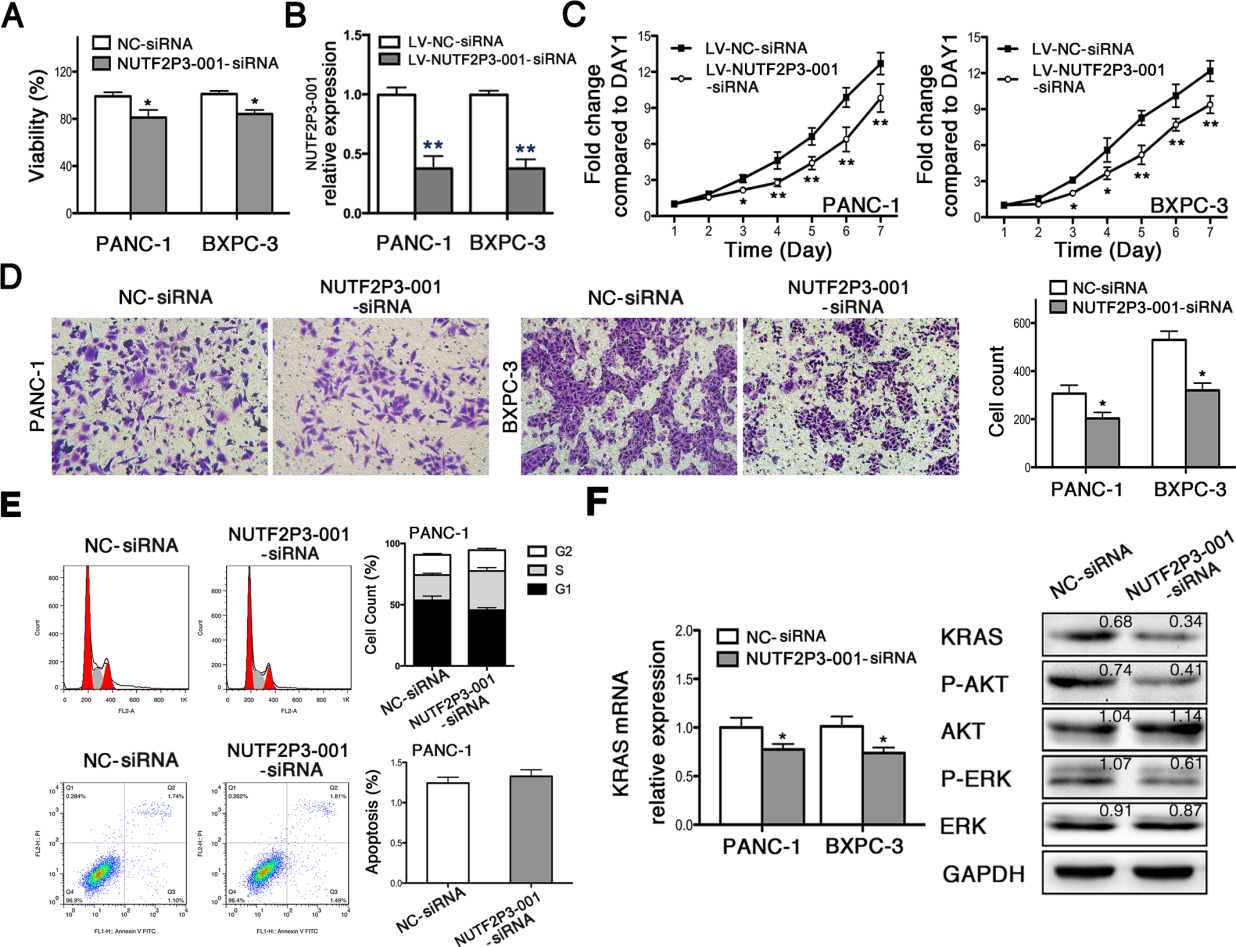
SiRNA-mediated knockdown of lncRNA-NUTF2P3-001 reduced viability, proliferation and invasive ability, while induced S phase arresting in pancreatic cancer cell (**A**) 72 h post transfection, MTT assays were performed and the results demonstrated that NUTF2P3-001-siRNA remarkably inhibited viability in pancreatic cancer cell. (**B**) 48 h post transfection, total RNA was extracted and qRT-PCR was used to identify the efficiency of transfection with lentivirus containing NUTF2P3-001-siRNA sequence (LV-NUTF2P3-001-siRNA) in pancreatic cancer cell. (**C**) Proliferation of pancreatic cancer cell was measured by MTT assays for 7 days observation. Compromised growth rate was observed in LV-NUTF2P3-001-siRNA transfected groups. (**D**) The capacity of invasion was identified by Transwell assay 48 h after transfection. (**E**) The cell cycle distribution and apoptosis were measured by flow cytometry in PANC-1 cells. The results showed that NUTF2P3-001-siRNA induced significant S-phase arrest but without significant effect on apoptosis. (**F**) NUTF2P3-001-siRNA obviously decreased KRAS mRNA expression both in PANC-1 and BXPC-3 cell lines. KRAS and its downstream proteins in PANC-1 treated with NUTF2P3-001-siRNA were tested by western blot. KRAS, p-AKT/AKT and p-ERK/ERK were significantly downregulated. Relative intensity value is marked. All data were presented as means ± SD of at least three independent experiments. The *p*-value represents the comparison between groups (*p < 0.05, **p < 0.01).

Since lncRNA-NUTF2P3-001 was upregulated in pancreatic cancer samples, we downregulated lncRNA-NUTF2P3-001 with lncRNA-NUTF2P3-001-siRNA (NUTF2P3-001-siRNA) to further identify its functional roles in pancreatic cell lines PANC-1 and BXPC-3. Three siRNA sequences were designed and the most effective one was chosen to fulfill the following experiment (Supplementary Figure 3A). After downregulated with NUTF2P3-001-siRNA, the viability of pancreatic cancer cell was significantly reduced (Figure 2A). Moreover, the proliferative ability of pancreatic cancer cell was remarkably inhibited after treated with lentivirus containing NUTF2P3-001-siRNA (LV-NUTF2P3-001-siRNA) (Figure 2B, 2C). Meanwhile, NUTF2P3-001-siRNA transfected cells were less observed on the membrane of lower chamber compared to NC-siRNA transfected cells in invasion assay (Figure 2D). Compared to NC-siRNA cells, the lncRNA-NUTF2P3-001 knockdown resulted in the accumulation of PANC-1 cells in the S-phase of cell cycle, while without effect on apoptosis (Figure 2E). More importantly, downregulation of KRAS and its downstream proteins, p-AKT and p-ERK (Figure 2F), were detected after NUTF2P3-001-siRNA transfection, indicating that the lncRNA-NUTF2P3-001 acted as promoter in pancreatic cancer *in vitro* and might play a role via regulating KRAS and its downstream pathways. Despite being statistically significant, differences on some functional assays and expression levels of KRAS signaling elements are moderate might due to the limited efficiency of siRNA-mediated silencing.

According to recent researches, large fraction of the lncRNAs present in mammalian cells is found to localize within cell nucleus and some of them participate in regulation of key nuclear processes. However, in general the efficiency of siRNA-mediated depletion of nuclear-retained RNAs is low due to the fact that the siRNA machinery is located mainly in the cytoplasm of mammalian cells [30, 31]. Hence, our siRNA efficiency of lncRNA-NUTF2P3-001 was moderated despite being statistically significant, for which reason that differences on functional assays and expression levels of KRAS signaling elements were limited.

### Overexpression of lncRNA-NUTF2P3-001 promotes viability and invasion in pancreatic cancer cell, accompanying with upregulated KRAS expression

After overexpression with pcDNA-NUTF2P3-001, the viability of pancreatic cancer cell was significantly promoted (Supplementary Figure 4B). Moreover, pcDNA-NUTF2P3-001 obtained much stronger invasive capacity compared to that of corresponding negative control (Supplementary Figure 4C). Compared to pcDNA-NC cells, the pcDNA-NUTF2P3-001 resulted in the compromised accumulation of PANC-1 cells in the S-phase of cell cycle (Supplementary Figure 4D). More importantly, upregulation of KRAS and activated downstream proteins, p-AKT and p-ERK (Supplementary Figure 4E), were detected after NUTF2P3-001 overexpression in PANC-1 cells, indicating that the lncRNA-NUTF2P3-001 promoted pancreatic cancer progression *in vitro* via regulating KRAS and its downstream pathways on the other hand.

### LncRNA-NUTF2P3-001 regulates KRAS expression through competitively binding with miR-3923

**Figure 3:**
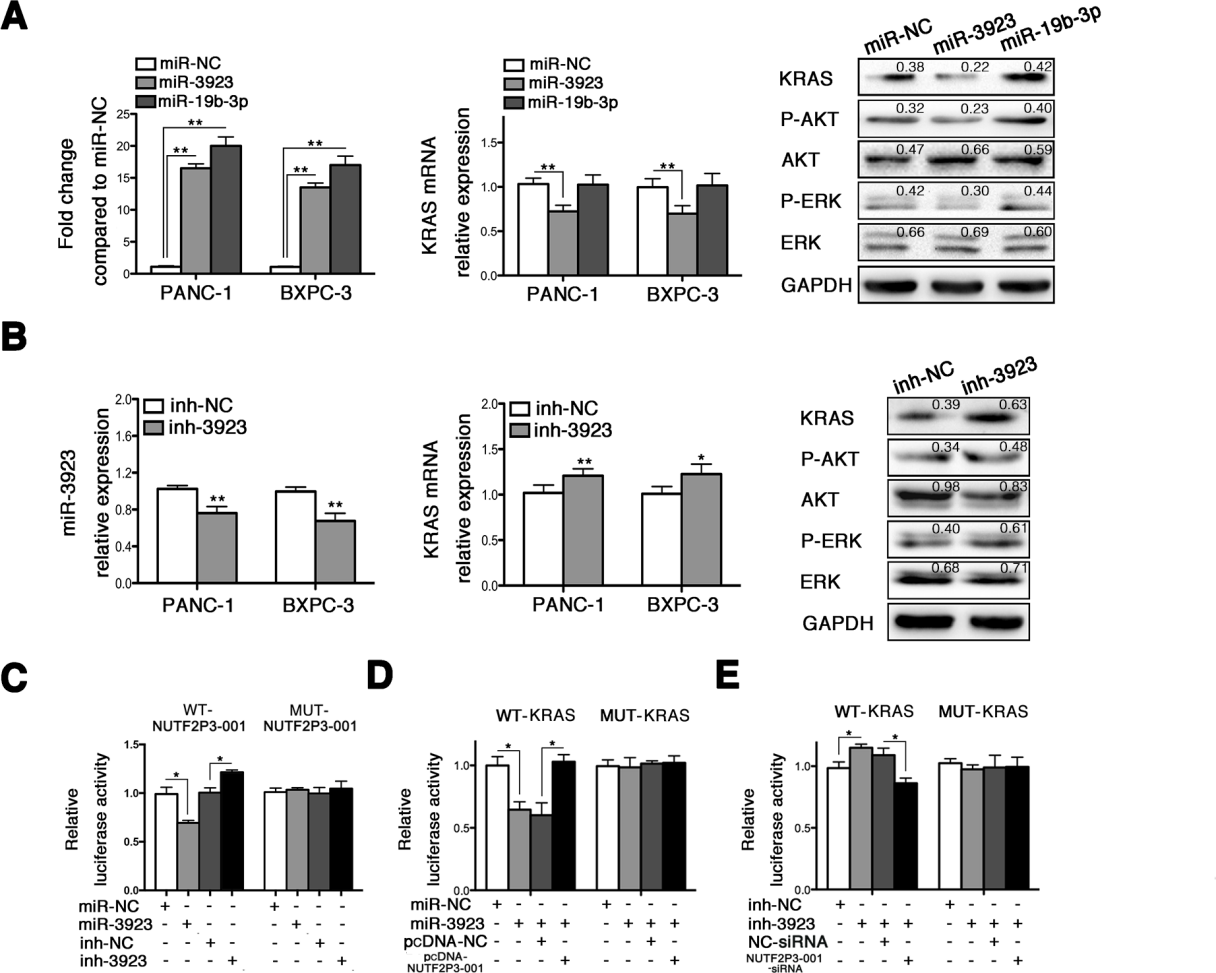
LncRNA-NUTF2P3-001 and 3′UTR of KRAS mRNA could competitively bind with miR-3923 (**A**) The expression of miR-3923 and miR-19b was remarkably increased after transfection with miR-3923 mimics (miR-3923, 50 nM) and miR-19b-3p mimics (miR-19b-3p, 50 nM) respectively, and the miR-NC was designated as value of 1. Only miR-3923 but not miR-19b-3p could decrease KRAS mRNA expression. The expression of KRAS and downstream proteins were detected in PANC-1 treated with miR-3923. MiR-3923 leaded to downregulated KRAS, p-AKT/AKT and p-ERK/ERK in PANC-1 cells. Relative intensity value is marked. (**B**) MiR-3923 was obviously decreased by transfection with miR-3923 inhibitor (inh-3923, 100 nM) and further lead to elevated level of KRAS in mRNA level. The expression of KRAS and downstream proteins were detected in PANC-1. Enhanced KRAS, elevated p-AKT/AKT and p-ERK/ERK were detected in PANC-1 cells. Relative intensity value is marked. (**C**) NUTF2P3-001 luciferase activity assays. Wide type or mutant NUTF2P3-001 was co-transfected with miR-3923 (50 nM) or inh-3923 (100 nM), respectively. MiR-3923 repressed, but inh-3923 enhanced the luciferase activity of the WT-NUTF2P3-001 reporter which including wild type sequence of NUTF2P3-001. There was no obvious change of the luciferase activity for the MUT-NUTF2P3-001 reporter which containing mutant NUTF2P3-001 sequence. (**D**) KRAS 3′UTR luciferase activity assays. Overexpression of NUTF2P3-001 with pcDNA-NUTF2P3-001 (0.2 μg per well in 96-well plate) rescued the luciferase activity, which repressed by the transfection with miR-3923 (50 nM) in the WT-KRAS but not in the MUT-KRAS reporter. (**E**) Downregulation of NUTF2P3-001 with NUTF2P3-001-siRNA (50 nM) reversed the luciferase activity, which enhanced by the transfection with inh-3923 (100 nM) in the WT-KRAS but not in MUT-KRAS reporter. The normalized luciferase activity in the control group was set to 1. All data were presented as means ± SD of at least three independent experiments. The *p*-value represents the comparison between groups (*p < 0.05, **p < 0.01).

We further identified the underlying regulative mechanisms of lncRNA-NUTF2P3-001 on KRAS expression. The miRNAs target prediction algorithm suggested that both lncRNA-NUTF2P3-001 and the 3′UTR of KRAS mRNA had potential binding sites for miR-3923 or miR-19b-3p (Supplementary Figure 3B). However, only miR-3923 but not miR-19b-3p could negatively regulate the expression of KRAS and corresponding downstream proteins (Figure 3A), which was further verified by the results that inhibition of miR-3923 upregulated KRAS pathway. We further validated the direct binding of lncRNA-NUTF2P3-001 and 3′UTR of KRAS mRNA with miR-3923 in pancreatic cancer cell by dual luciferase reporter assay. The results demonstrated that the miR-3923 mimics remarkably reduced, but the miR-3923 inhibition increased luciferase activities of the reporter plasmid containing the potential binding sequence of 3′UTR of KRAS mRNA or lncRNA-NUTF2P3-001 (wild type, WT), but without obvious changes in the reporter plasmid containing mutated sequence (mutant type, MUT) (Figure 3C). Moreover, co-transfection of lncRNA-NUTF2P3-001 could rescue the decreased luciferase activity of WT-KRAS treated with miR-3923 mimics (Figure 3D). On the contrary, the luciferase activities of the WT-KRAS were enhanced by miR-3923 inhibition, which could be reversed by NUTF2P3-001-siRNA respectively (Figure 3E). These data illustrated that lncRNA-NUTF2P3-001 directly regulated KRAS expression through competitively binding with miR-3923 as a miRNAs sponge.

### The expression of miR-3923 is significantly downregulated in pancreatic cancer but not in chronic pancreatitis tissues

To further reveal the function of miR-3923 in pancreatic cancer, we tested the expression of miR-3923 in pancreatic cancer and chronic pancreatitis tissues by qRT-PCR. Compared with the noncancerous pancreatic tissues, obvious reduced miR-3923 was observed in pancreatic cancer but not chronic pancreatitis specimens (Supplementary Figure 5A). Nevertheless, no statistical correlation was demonstrated in expression of miR-3923 with KRAS and lncRNA-NUTF2P3-001 in pancreatic tissues (Supplementary Figure 5B), which further implied that the overexpression of KRAS might depend on not only the decreased miR-3923 expression but also the competitive binding of overexpressed lncRNA-NUTF2P3-001.

### Overexpression of miR-3923 simulates the roles of NUTF2P3-001-siRNA on pancreatic cancer cell

To further identify the pivotal role of miR-3923 in crosstalk between lncRNA-NUTF2P3-001 and KRAS mRNA, the biological behaviors of pancreatic cancer cell were observed after miR-3923 mimics transfection. As shown in Supplementary Figure 6A, miR-3923 mimics significantly reduced the viability of PANC-1 and BXPC-3. Moreover, the proliferation of PANC-1 and BXPC-3 was also significantly inhibited after transfection with lentivirus containing miR-3923 (LV-miR-3923) (Supplementary Figure 6B, 6C). Meanwhile, miR-3923 mimics remarkably decreased the invasive ability of pancreatic cancer cell (Supplementary Figure 6D), as well as leaded to increased S-phase arresting in PANC-1 (Supplementary Figure 6E). Similar to lncRNA-NUTF2P3-001, miR-3923 mimics did not induce obvious apoptosis of PANC-1 (Supplementary Figure 6F). These results indicated that the miR-3923 mimics simulated the inhibitor effects of NUTF2P3-001-siRNA.

### The miR-3923 inhibitor rescues the inhibiting effects of LV-NUTF2P3-001-siRNA on pancreatic cancer cell

**Figure 4:**
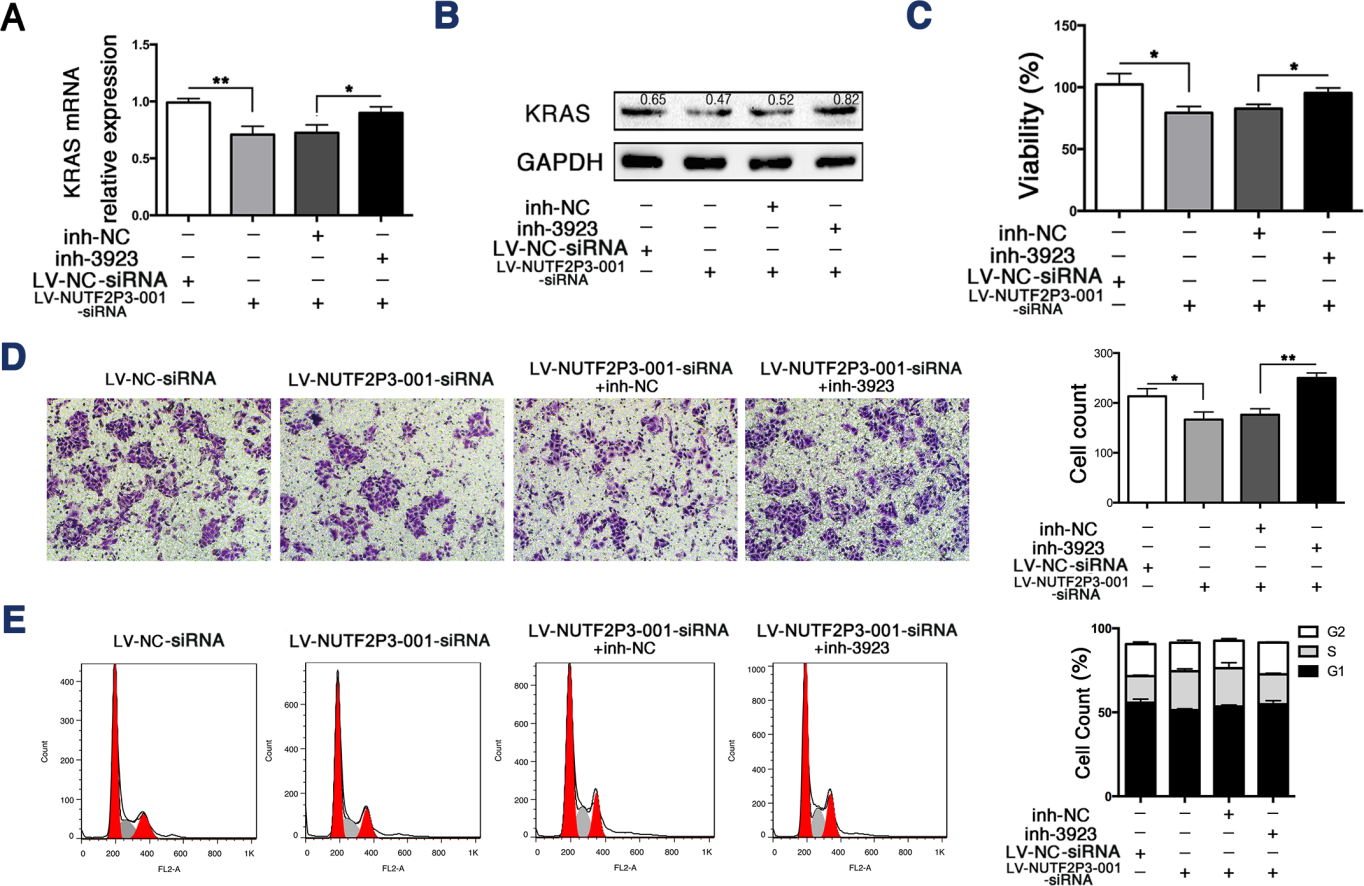
The miR-3923 inhibitor rescued the inhibiting effects of LV-NUTF2P3-001-siRNA on PANC-1 cells (**A**) The expression of KRAS of PANC-1 treated with LV-NUTF2P3-001-siRNA or inh-3923 (100 nM) was examined by qRT-PCR. (**B**) Western blot analysis was performed to identify KRAS expression in transfected PANC-1 cells. Transfection with LV-NUTF2P3-001-siRNA decreased the expression of KRAS, which was further rescued by co-transfection with miR-3923 inhibitor. Relative intensity value is marked. (**C**) The inh-3923 reversed the inhibition of LV-NUTF2P3-001-siRNA on the viability of PANC-1 cells. (**D**) Matrigel invasion assays were performed to measure the invasive ability of PANC-1 cells treated with LV-NUTF2P3-001-siRNA and inh-3923 co-transfection. Results were quantified on the right. (**E**) Cell cycle distribution was measured by flow cytometry with PI staining. Increased S-phase proportion was observed in LV-NUTF2P3-001-siRNA treated group and further decreased after inh-3923 transfection. Contribution of different phases was shown on the right. All data were presented as means ± SD of at least three independent experiments. The *p*-value represents the comparison between groups (*p < 0.05, **p < 0.01,).

To further identify the interaction between miR-3923 and lncRNA-NUTF2P3-001 in the regulation of KRAS expression, we transfected miR-3923 inhibitor into PANC-1 cells treated by LV-NUTF2P3-001-siRNA. The data revealed that miR-3923 inhibitor significantly promoted KRAS expression (Figure 4A, 4B), viability (Figure 4C) and invasive ability (Figure 4D) of LV-NUTF2P3-001-siRNA transfected PANC-1 cells. Similarly, the S-phase arrest of LV-NUTF2P3-001-siRNA transfected PANC-1 cells was also rescued by transfection with miR-3923 inhibitor (Figure 4E). These results further validated the inhibiting roles of NUTF2P3-001-siRNA on pancreatic cancer cell depended on enhancing the binding of miR-3923 with KRAS.

### Both downregulated lncRNA-NUTF2P3-001 and overexpressed miR-3923 inhibit tumor growth and hepatic metastasis *in vivo*

**Figure 5:**
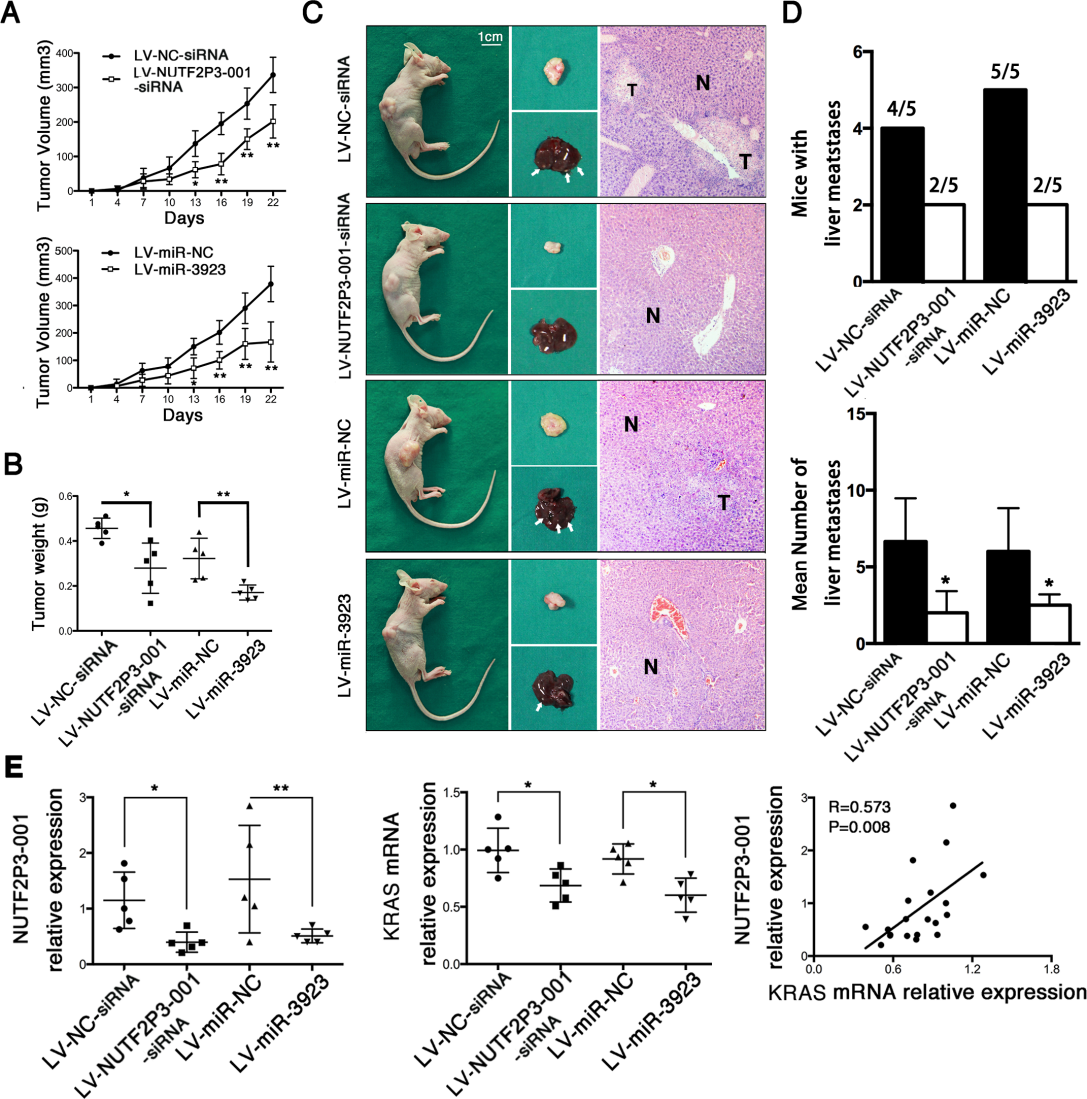
Both NUTF2P3-001-siRNA and miR-3923 could inhibit tumor growth and metastasis of pancreatic cancer xenograft in nude mice (**A**) Stable transfected PANC-1 cells was injected subcutaneously into the right flank of 3-week-old male BALB/c nude mice (*n* = 5 per group). The tumor volumes were measured every 3 days (tumor volume = length × width^2^/2). (**B**) Nude mice were sacrificed and the weights of tumor in LV-NUTF2P3-001-siRNA, LV-NC-siRNA, LV-miR-3923 and LV-miR-NC groups are measured respectively. (**C**) Nude mice, corresponding tumors, liver tissues and HE staining slices after 3 weeks inoculation. T stands for metastasis and N for normal liver tissues. (**D**) The mice with liver metastases and the mean number of liver metastases were quantified. Downregulated NUTF2P3-001 and overexpressed miR-3923 results in lower incidence of liver metastases (4/5 vs 2/5, 5/5 vs 2/5) and less number of metastases in liver (6.9 vs 2.2, 6.1 vs 2.5). (**E**) The relative expression of NUTF2P3-001 and KRAS in the implanted pancreatic cancer tumors of each group. The data showed that the expression of NUTF2P3-001 is significantly positive correlated with that of KRAS (*r* = 0.573, *p* = 0.008). All data were presented as means ± SD of at least three independent experiments. The *p*-value represents the comparison between groups (*p < 0.05, **p < 0.01,).

In order to further validate the effect of lncRNA-NUTF2P3-001 and miR-3923 on growth and invasion of pancreatic cancer *in vivo*, the PANC-1 cells transfected with LV-NUTF2P3-001-siRNA, LV-NC-siRNA, LV-miR-3923 and LV-miR-NC were injected subcutaneously into BALB/c athymic nude mice of 3-week-old (*n* = 5 per group) respectively. Compared with associated NC group, the growth rate (Figure 5A) and weight of tumors in LV-NUTF2P3-001-siRNA and LV-miR-3923 treated model were both significantly reduced (Figure 5B). Moreover, both the number of mice with liver metastases and the number of liver metastasis in mice were both obviously less in LV-NUTF2P3-001-siRNA and LV-miR-3923 group compared to corresponding NC groups (Figure 5C, 5D). Afterwards, implanted tumors of both LV-NUTF2P3-001-siRNA and LV-miR-3923 groups showed significantly decreased lncRNA-NUTF2P3-001 and KRAS expression, which also strongly positive correlated with each other (Figure 5E).

### The lncRNA-NUTF2P3-001 is responsively upregulated in hypoxia as a direct transcriptional target of HIF-1α

**Figure 6:**
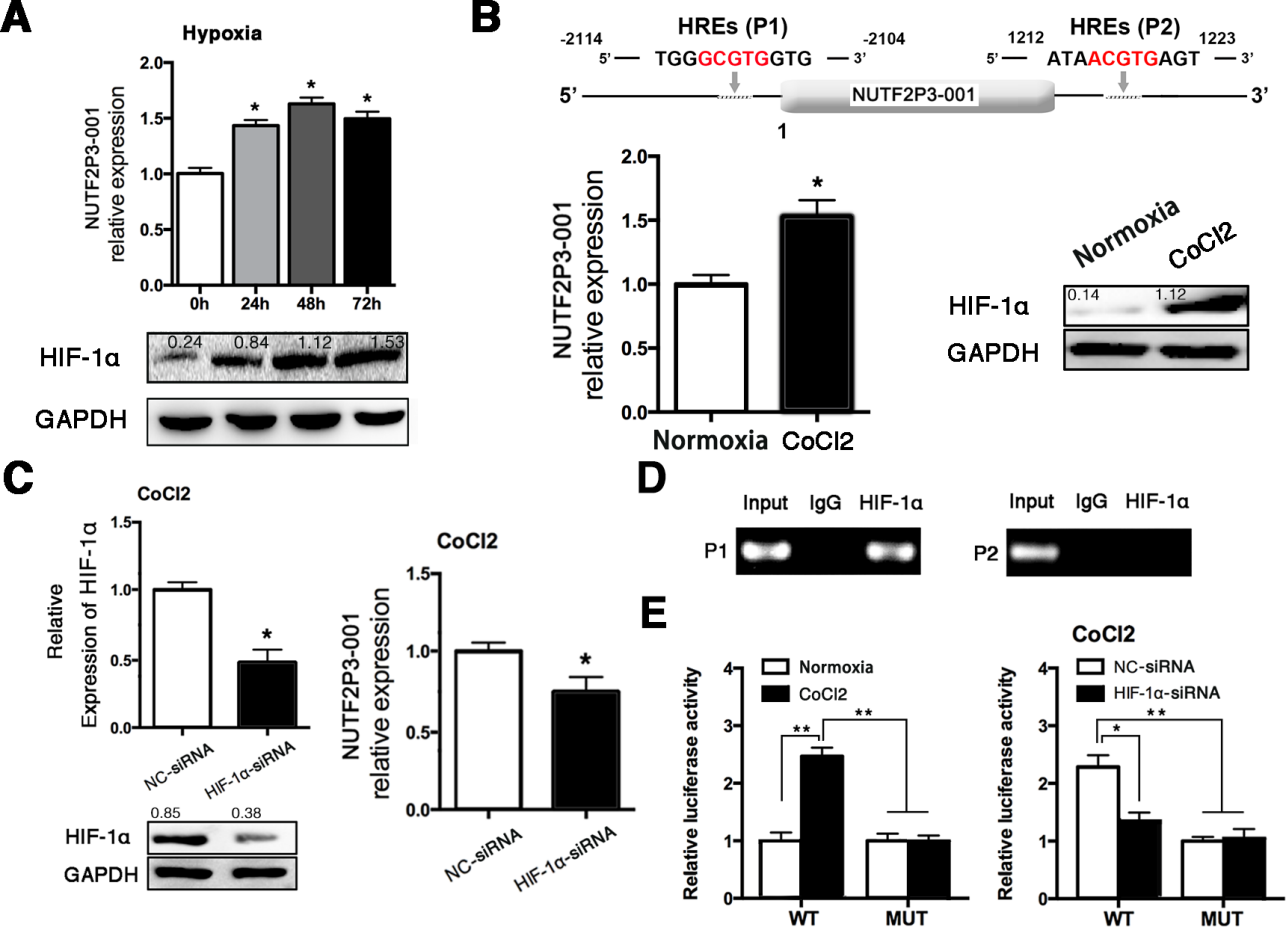
Hypoxia treatment upregulated the expression of lncRNA-NUTF2P3-001 in PANC-1 cells (**A**) We tested the expression of NUTF2P3-001 in PANC-1 cells treated with hypoxia for 24 h, 48 h and 72 h (upper). Results of western blot demonstrated boosted HIF-1α expression in PANC-1 cells after hypoxia exposure (lower). Hypoxia represents cells cultured with 1% O_2_, 5% CO_2_ and 94% N_2_. (**B**) Two putative HREs (G/ACGTG) were found in the promoter of NUTF2P3-001 gene (upper). After treated with CoCl_2_ (100 μM) for 48 h, NUTF2P3-001 was detected in PANC-1 by qRT-PCR and HIF-1α protein was evaluated by western blot (lower). Relative intensity value is marked. (**C**) HIF-1α level was evaluated by western blot and qRT-PCR after HIF-1α-siRNA transfection (left). Expression of NUTF2P3-001 was tested by qRT-PCR in CoCl_2_ treated PANC-1 after transfection with HIF-1α-siRNA (50nM) (right). The upregulation of NUTF2P3-001 caused by CoCl_2_ treatment could be rescued by HIF-1α-siRNA. (**D**) ChIP assays were performed to verify the binding between HIF-1α and HREs. Primer 1 (P1) and primer 2 (P2) were designed to detect HREs1 and HREs2, respectively. PCR products were separated by gel electrophoresis on 2% agarose gel. (**E**) Exposure of CoCl_2_ resulted in significantly enhanced active effects of HIF-1α on HREs1 in NUTF2P3-001 promoter, compared with that of normoxia condition (left). Dual luciferase assay was performed, the activated HREs1 caused by CoCl_2_ treatment could be rescued by HIF-1α-siRNA (right). All data were presented as means ± SD of at least three independent experiments. The *p*-value represents the comparison between groups (*p < 0.05, **p < 0.01,).

In order to reveal the mechanism for the upregulation of lncRNA-NUTF2P3-001 in pancreatic cancer tissues, PANC-1 cells were cultured under hypoxia up to 72 h. As shown in Figure 6A, both HIF-1α and lncRNA-NUTF2P3-001 were significantly upregulated in hypoxia induction. Meanwhile, miR-3923 was not decreased as we expected and even elevated in hypoxia treatment (data not shown). Since two HREs were found in the promoter of lncRNA-NUTF2P3-001 when we inspected the genomic sequence of the lncRNA-NUTF2P3-001 gene (Figure 6B upper), we next explored whether HIF-1α could regulate lncRNA-NUTF2P3-001 expression at the transcriptional level. After treated cells with CoCl_2_, which stabilizes the HIF-1α protein, significantly lncRNA-NUTF2P3-001 expression was upregulated companying with increased HIF-1α protein (Figure 6B lower). Furthermore, three HIF-1α-siRNA sequences were designed and the most effective one was chosen to further evaluate the effects of HIF-1α on lncR-NUTF2P3-001 expression (Supplementary Figure 7A). The HIF-1α-siRNA obviously decreased the expression of lncRNA-NUTF2P3-001 under CoCl_2_ treatment (Figure 6C). As shown in Figure 6D upper, the ChIP assay further validated the specific binding of HIF-1α and HRE1 in promoter of lncRNA-NUTF2P3-001 gene but not in HRE2. In addition, we further evaluated whether HIF-1α could regulate the expression of lncRNA-NUTF2P3-001 by activating the HRE1 within the lncRNA-NUTF2P3-001 gene promoter by luciferase reporter assay. DNA fragments containing wild-type or mutant HRE1 were inserted into the promoter region of a dual luciferase reporter plasmid. As was expected, luciferase density in the wild-type was remarkably induced by CoCl_2_ treatment, compared with that in normoxia condition. Moreover, this hypoxia-induced luciferase expression was greatly inhibited followed by knockdown of HIF-1α (Figure 6E). These data demonstrate that lncRNA-NUTF2P3-001 was transcriptionally upregulated by HIF-1α in hypoxia.

## DISCUSSION

Recent research has revealed that lncRNAs play pivotal roles in tumorigenesis of cancers including prostate cancer, melanoma, glioblastoma and oesophageal adenocarcinoma [5, 6, 32, 33]. However, the knowledge about the function of lncRNAs in tumorigenesis of pancreatic cancer is far from defined. Therefore, it is extremely valuable to identify the functional lncRNA and corresponding molecular mechanism on tumorigenesis of pancreatic cancer. The meaningful novel finding of present study is that hypoxia-induced lncRNA-NUTF2P3-001 functions as promoter through depressing the inhibition of miR-3923 on KRAS expression in pancreatic cancer.

The microarray results showed that the lncRNA-NUTF2P3-001 was one of the significantly increased lncRNAs, which was positively associated with KRAS mRNA expression both in pancreatic cancer and chronic pancreatitis samples. Meanwhile, the pancreatic cancer patients with higher lncRNA-NUTF2P3-001 expression demonstrated significant metastasis and worse prognosis. Moreover, the knockdown of lncRNA-NUTF2P3-001 significantly decreased KRAS expression, proliferation and invasive ability of pancreatic cancer cell both *in vitro* and *in vivo*. These results clarify that lncRNA-NUTF2P3-001 can function as promoter in pancreatic cancer by derepressing the inhibition of miR-3923 on KRAS expression.

Since KRAS and lncRNA-NUTF2P3-001 locate at different chromosome, we speculated that lncRNA-NUTF2P3-001 might regulate KRAS expression by acting as ceRNAs to competitively combine with specific miRNA. The target prediction algorithm indicates the potential existence of a specific crosstalk between the lncRNA-NUTF2P3-001 and KRAS through competitive binding with miR-3923 or miR-19b-3p. Interestingly, only miR-3923 but not miR-19b-3p overexpression could significantly inhibit KRAS expression and decrease the fluorescence density of dual-luciferase assay. This data implies that miR-3923 is the only point, which is competitively banded by lncR-NUTF2P3-001 and KRAS mRNA.

Furthermore, the present study displayed that miR-3923 overexpression significantly inhibited proliferation and invasion of pancreatic cancer cell both *in vivo* and *in vitro*, which dramatically simulating the suppressive effects of NUTF2P3-001-siRNA. On the other hand, the miR-3923 inhibition remarkably rescued the suppression of NUTF2P3-001-siRNA on pancreatic cancer cell. Although miR-3923 is significantly decreased in pancreatic cancer tissue, data does not show statistical correlation between miR-3923 and KRAS expression. This result further indicates that the overexpression of KRAS may attribute to not only the miR-3923 downregulation, but more rely on the competitive binding of overexpressed lncRNA-NUTF2P3-001. Also, miR-3923 might act as an inhibitor in the tumorigenesis of pancreatic cancer by mediating the regulation of lncRNA-NUTF2P3-001 on KRAS expression.

As a result of hypoxia in pancreatic cancer, hypoxia inducible factor-1 (HIF-1) is significantly overexpressed in pancreatic cancer tissue and cell [34, 35]. HIF-1 is a heterodimer, including an α subunit and a β subunit^36,37^. In response to hypoxia, HIF-1α, which determines HIF-1 activity, is the predominant mediator in regulating downstream genes. This active transcription factor can combine with the hypoxia-response elements (HREs) and then enhances target genes expression, including lncRNAs [26, 27]. Xue M et al. demonstrated that lncRNAs-UCA-1, upregulated by HIF-1, facilitated bladder cancer cell invasion in hypoxia [27]. Yang F et al. displayed that a positive feedback loop between HIF-1α and lincRNA-p21, which promoting glycolysis under hypoxia [38]. On the contrary, the other researches demonstrated that hypoxia repressed expression of lncRNA-LET by reducing the histone acetylation-mediated modulation of the lncRNA-LET promoter region [39]. Since the sequence analysis shows there are two potential putative HREs (5′-GCGTG-3′) within the promoter of the lncRNA-NUTF2P3-001, we presume that the HIF-1α might transcriptionally regulate lncRNA-NUTF2P3-001 expression by binding with HRE of promoter area. Our data showed that lncRNA-NUTF2P3-001 in pancreatic cancer cell was elevated under hypoxia or CoCl_2_, while decreased with the HIF-1α-siRNA. Moreover, the combination and activity of HIF-1α with promoter of lncRNA-NUTF2P3-001 was further confirmed by the ChIP and luciferase reporter assay respectively. Therefore, all of these data intensively suggests that lncRNA-NUTF2P3-001 is transcriptionally regulated by HIF-1α in hypoxia of pancreatic cancer.

In summary, our work highlights the importance of the complicated miRNAs-lncRNA network in modulating the cellular response to hypoxic microenvironment of pancreatic cancer. Furthermore, our findings demonstrate the crucial role of lncRNA-NUTF2P3-001 mediated translational desuppression in KRAS expression and the therapeutic potential of targeting this pathway in pancreatic cancer.

## MATERIALS AND METHODS

### Patients and tissue samples

Tissue samples were obtained from patients undergoing surgery at the Pancreatic Disease Institute, Union Hospital (Wuhan, China), from July 2011 to July 2012. During these procedures, 30 PC, 10 CP and 30 NP were collected. Those patients were treated with pancreatectomy or palliative surgery including implantation of I^125^ seeds as well as choledochojejunostomy and gastroenterostomy, depending on the National Comprehensive Cancer Network (NCCN) guideline for pancreatic cancer (version 1. 2011) [40]. 30 NP samples were collected from surrounding tissues of serous cystadenoma or insulinoma, which were pathologically verified as NP tissue. Moreover, 10 CP samples were obtained from surgical resection of chronic pancreatitis patients. None of the PC patients had received chemotherapy or radiotherapy before the surgical excision. Appropriate Institutional Review Board approval and informed consent were received prior to the start of this study. Tissue samples were stored at −80°C immediately upon obtained.

### Cell culture

Cells were incubated in 5% CO_2_ at 37°C and grown in complete medium, which was composed of 90% RPMI-1640 (HyClone), 10% fetal bovine serum (HyClone) and 100 U/mL penicillin and 100 mg/ml streptomycin (HyClone). Pancreatic cancer cell lines, PANC-1 and BXPC-3, were bought from American Type Culture Collection (ATCC, USA). They were tested and authenticated for genotypes by DNA fingerprinting. These cell lines were passaged for less than 6 months after resuscitation, and no re-authorization was done. In order to build the hypoxia model, cells were cultured with 1% O_2_, 5% CO_2_ and 94% N_2_ or treated with CoCl_2_ (100 μM) [41]. Total RNA was collected at the time point of 0 h, 24 h, 48 h and 72 h.

### Microarrays

Total RNA was extracted from tissue samples and cleaned up firstly. Then prepare labeling reaction by using Quick Amp Labeling Kit, One-Color (Agilent). These cDNA samples were then cleaned and labeled in accordance with the Agilent Gene Expression Analysis protocol using Low Input Quick-Amp Labeling Kit, one-color (Agilent p/n 5190-0442). These labeled cDNA samples were purified and further used as probes to hybridize to microarrays for 17 h at 65°C using an Agilent Gene Expression Hybridization Kit (Agilent p/n 5188-5242) in hybridization chamber gasket slides (Agilent p/n G2534-60003). After hybridization, the microarrays were washed and scanned with an Agilent microarray scanner (Agilent p/n G2565BA).

Markov cluster algorithm (MCL) was used to identify co-expressed functional module in the CNC network. Moreover, we identified well-established miRNAs that bind lncRNAs by using an algorithm named miRcode that predicts putative microRNA binding sites in lncRNAs using criteria such as seed complementarity and evolutionary conservation.

### Transfection

LncRNA-NUTF2P3-001-siRNA (NUTF2P3-001-siRNA), lncRNA-NUTF2P3-001 pcDNA3.1 plasmid (pcDNA-NUTF2P3-001), miR-3923 mimics (miR-3923), miR-19b-3p mimics (miR-19b-3p), miR-3923 inhibitor (inh-3923), HIF-1α-siRNA and corresponding negative control were purchased from Ribobio Co. (Guangzhou, China). NUTF2P3-001-siRNA, HIF-1α-siRNA, miR-19b-3p and miR-3923 were transfected with Lipofectamine 2000 (Invitrogen) at a final concentration of 50 nM, while the final concentration for inh-3923 was 100 nM and all plasmids were transfected with 0.2 μg for 96 well plate and 1.6 μg for 12 well plate. The effects of the transient transfection were confirmed by quantitative real-time PCR analysis. We extracted protein and total RNA 48 h post-transfection. All siRNA sequences and NUTF2P3-001 clone sequence in pcDNA3.1 are shown in Supplementary Table 1.

Lentivirus vector (GV115) containing NUTF2P3-001-siRNA sequence (LV-NUTF2P3-001-siRNA), LV-miR-3923 and corresponding negative control (LV-NC-siRNA and LV-miR-NC) were purchased from GeneChem Company (Shanghai, China). According to the results of preliminary experiment, MOI (multiplicity of infection) values of PANC-1 and BXPC-3 were 40 and 80 respectively. Cells were cultured in ENi.S. (Enhanced infection Solution) with Polybrene (5 μg/ml) and lentivirus. 12 hours later, the mix was changed with complete medium. At last, we identified the transfection efficiency by observing fluorescence under fluorescence microscope 3 days later.

### Western blot analysis

Western blot analysis has been done as described previously [42]. Antibodies for research were as follows: Rabbit anti-KRAS (1:500) was purchased from ImmunoWay (ImmunoWay Biotechnology, USA), rabbit anti-GAPDH (1:1000), anti-P-AKT (1:1000), anti-AKT (1:1000), anti-P-ERK (1:1000), anti-ERK (1:1000) were bought from CST (Cell Signaling Technology, Inc, USA), mouse anti-HIF-1α monoclonal (1:1000) were purchased from Abcam Biotechnology (Abcam, Cambridge, UK). Rabbit and mouse secondary antibodies (1:3000) were purchased from CST.

### Quantitative real-time reverse transcription polymerase chain reaction (qRT-PCR)

Total RNA was extracted from tissues and cells by using RNAiso Plus (TAKARA) according to the product description. QRT-PCR was used to check the expression level of mRNAs. All mRNAs and miRNAs were reverse transcribed according to the protocol of the PrimeScript^®^ RT Master Mix Perfect Real Time (TAKARA) and One Step PrimeScript^®^ miRNA cDNA Synthesis Kit (Perfect Real Time) (TAKARA), followed by qRT-PCR analysis with the SYBR Premix Ex Taq II (TAKARA) as the manufacturer's protocol. All reactions were performed in triplicate. The primers were shown in Table 2.

**Table 2:** Primers for qRT-PCR and ChIP assays

Targets	Sequences
NUTF2P3-001 forward	5′-GGAAGGACAGCAATGACA-3′
NUTF2P3-001 reverse	5′-AATAGGAACATCTGGTGGAA-3′
KRAS forward	5′-CCAGGCCTGCTGAAAATGAC-3′
KRAS reverse	5′-CCCTCCCCAGTCCTCATGTA-3′
GAPDH forward	5′-GAAGGTGAAGGTCGGAGTC-3′
GAPDH reverse	5′-GAAGATGGTGATGGGATT-3′
MiR-3923 forward	5′-AACTAGTAATGTTGGATTAGGG-3′
MiR-19b-3p forward	5′-TGTGCAAATCCATGCAAAACTGA-3′
U6 forward	5′-CTCGCTTCGGCAGCACA-3′
P1 forward	5′-GATACTCATCATCAGAACCCAC-3′
P1 reverse	5′-ACAACCTCCGCATACCAG-3′
P2 forward	5′-AGGGAGCTAACAGGTTGA-3′
P2 reverse	5′-AGTTGTCTCGGACTTGGA-3′

### MTT assay for cell viability and proliferation

After stable transfection, 2.5 × 10^3^ cells were seeded in new 96-well plate with complete medium, the viability was assessed after 2 days and the proliferation was observed for 7 days. Cells were cultured with 20 μl MTT (5 mg/ml) per well for 4 h in incubator. Then replaced the mix of MTT and medium with 150 μl dimethylsulfoxide (Sigma) each well. Finally, determined the absorbance by ELISA reader at 570 nm when the crystals were totally dissolved. All MTT assays were repeated three times in five replicates.

### Invasion assay

We maintain the cells' invasion capacity by using the Matrigel Invasion Chamber of pore size 8 μm (Corning, Fisher Scientific, UK). The chambers were coated with 1:9 diluted Matrigel (Sigma) for 4 hours, 5 × 10^4^ cells were placed in the upper chamber with 200 μl medium containing 0.1% serum. Then 700 μl medium containing 30% serum was placed in the lower chambers [43, 44]. Fixed and stained 2 days later, count the number of cells on the membrane of lower chamber under a microscope in nine fields.

### Cell apoptosis and cell cycle analysis

For cell apoptosis analysis, diluted the cells to 10^6^ per ml with 500ul binding buffer, followed the instruction protocol, cells were stained with Annexin V-FITC (5 μl) and PI (10 μl, 20 μg/ml) for 15 mins at 4 degrees. For cell cycle analysis, prior to treat with PI (50 μg/ml) and RNase-A (25 μg/ml) for 15 mins, cells were fixed in 70% cold ethanol at −20°C overnight. Cells were analyzed by a FACS Calibur flow cytometer (BD Biosciences) and data were analyzed with Flowjo software. All of these were repeated in duplicates.

### Luciferase activity assay

For KRAS mRNA 3′UTR luciferase reporter assays, luciferase reporter plasmid (100 ng) containing the potential binding sequence of 3′UTR of KRAS mRNA (wild type, WT) or mutated sequence (mutant type, MUT) were co-transfected into PANC-1 cells in 96-well plate with miR-3923 (50 nM), inh-3923 (100 nM), NUTF2P3-001-siRNA (50 nM), pcDNA-NUTF2P3-001 (0.2 μg), and corresponding NC respectively by using Lipofectamine 2000 (Invitrogen). For lncRNA-NUTF2P3-001 promoter luciferase reporter assays, luciferase reporter plasmid (100 ng) containing the potential HRE1 sequence (wild type, WT) or mutant sequence (mutant type, MUT) were co-transfected into PANC-1 cells in 96-well plate with HIF-1α-siRNA (50 nM) and corresponding NC. Luciferase activity assays were performed 48 hours after transfection (Promega, Madison, WI). Firefly luciferase activity was normalized to the corresponding renilla luciferase activity by using the Dual-Luciferase Reporter Assay System. All experiments were performed three times.

### ChIP assays

ChIP assays were performed by using EpiQuik^tm^ Chromatin Immunoprecipitation Kit and anti-HIF-1α (ab1, Abcam, Cambridge, UK) according to the manufacturer's instructions. Corresponding IgG was used as controls. The bound DNA fragments were subjected to PCR reactions using the primers in Table 2. PCR products were separated by gel electrophoresis on 2% agarose gel.

### Pancreatic cancer mouse models

Stable transfected PANC-1 cells (0.5 × 10^7^ in 200 μl PBS) were injected subcutaneously into the right flank of 3-week-old male BALB/c nude mice (*n* = 5 per group). The tumor size was measured every 3 days and calculated by the formula: tumor volume = length × width^2^/2 [45]. All nude mice were used following protocols approved by the Animal Care and Use Committee of Tongji Medical College of Huazhong University of Science and Technology.

### Statistical analysis

All results are presented as means ± standard deviation (SD). Unpaired *t* tests were used to statist the comparisons between groups. The relationships between lncRNA-NUTF2P3-001 expression and clinical characteristics were analyzed by *χ*^2^ tests. Pancreatic cancer patient survival was analyzed by log-rank test. The relationships among lncRNA-NUTF2P3-001, miR-3923 and KRAS expression were explored by Spearman's correlation. When *P* < 0.05, the values were considered to be significantly different. All data was analyzed by using SPSS 13.0 software.

## SUPPLEMENTARY MATERIALS FIGURES AND TABLE


