# Supplementary material for: Hypoxia-induced lncRNA-NUTF2P3-001 contributes to tumorigenesis of pancreatic cancer by derepressing the miR-3923/KRAS pathway
Source: Oncotarget. 2016 Jan 7;7(5):6000–14. doi: 10.18632/oncotarget.6830 (PMC4868736; doi:10.18632/oncotarget.6830)
Supplement: Supplementary file 1 [file oncotarget-07-6000-s001.pdf]

Hypoxia-induced lncRNA-NUTF2P3-001 contributes to tumorigenesis of pancreatic cancer by derepressing the miR-3923/KRAS pathway

Supplementary Materials

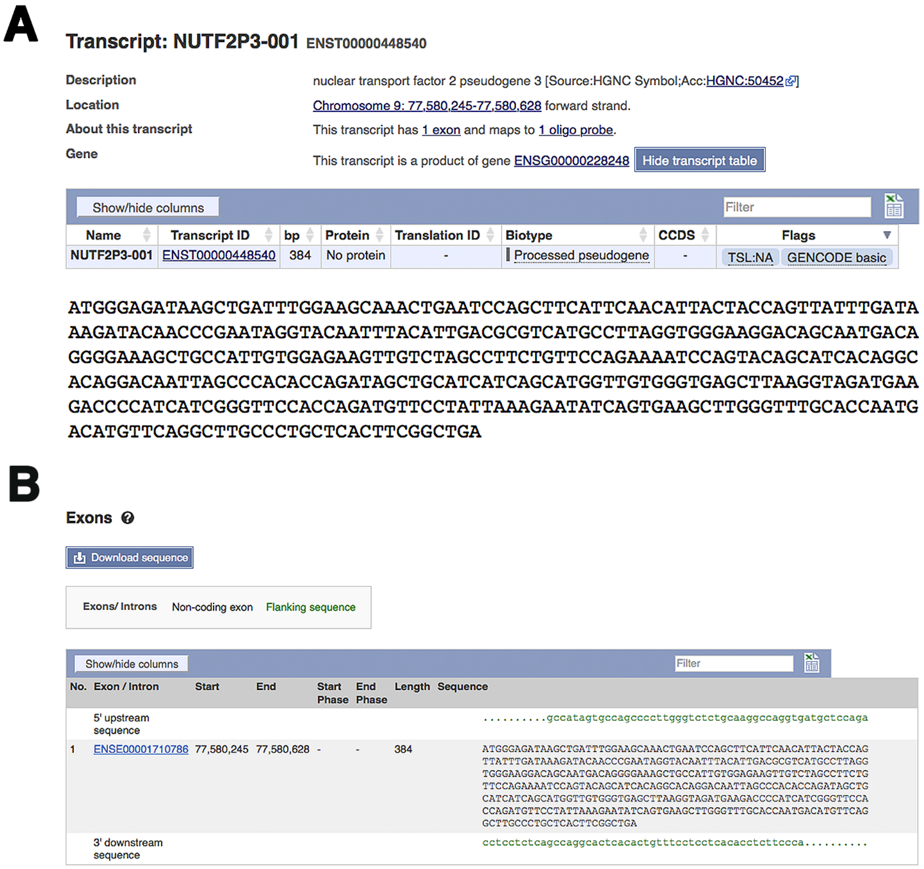

Supplementary Figure S1: The information of lncRNA-NUTF2P3-001. (**A**) The description, location and sequence of NUTF2P3-001. (**B**) The exon of this transcript.

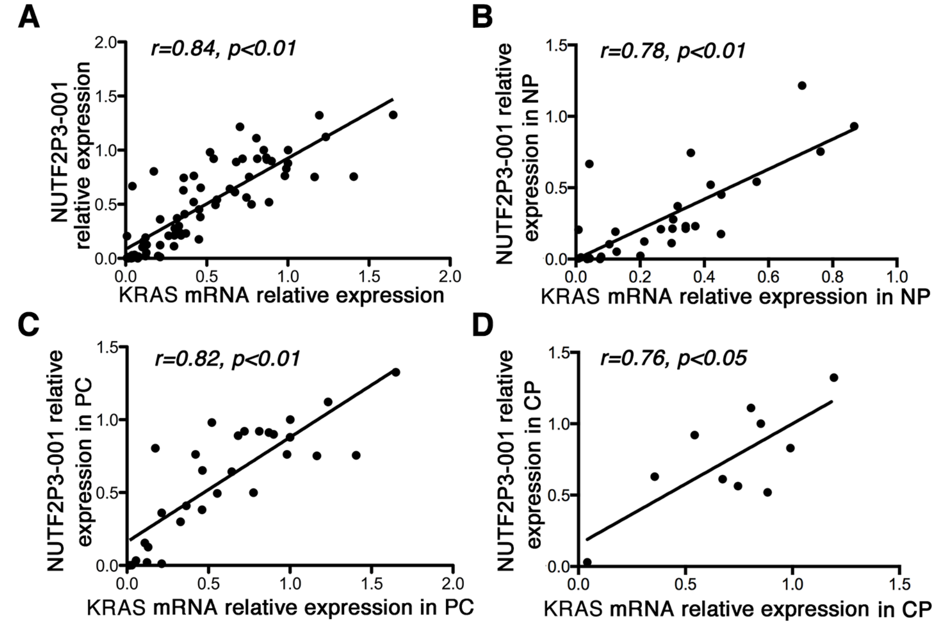

Supplementary Figure S2: The correlation between expression of lncR-NUTF2P3-001 and KRAS mRNA in different pancreatic tissues. (**A**) The correlations between NUTF2P3-001 and KRAS mRNA in pancreatic tissues. (**B**) The correlations between NUTF2P3-001 and KRAS mRNA level in noncancerous pancreatic tissue (NP). (**C**) The correlations between NUTF2P3-001 and KRAS mRNA level in pancreatic cancer (PC). (**D**) The correlations between NUTF2P3-001 and KRAS mRNA level in chronic pancreatitis (CP). The *p*-value represents the comparison between groups (*$p < 0.05$, **$p < 0.01$).

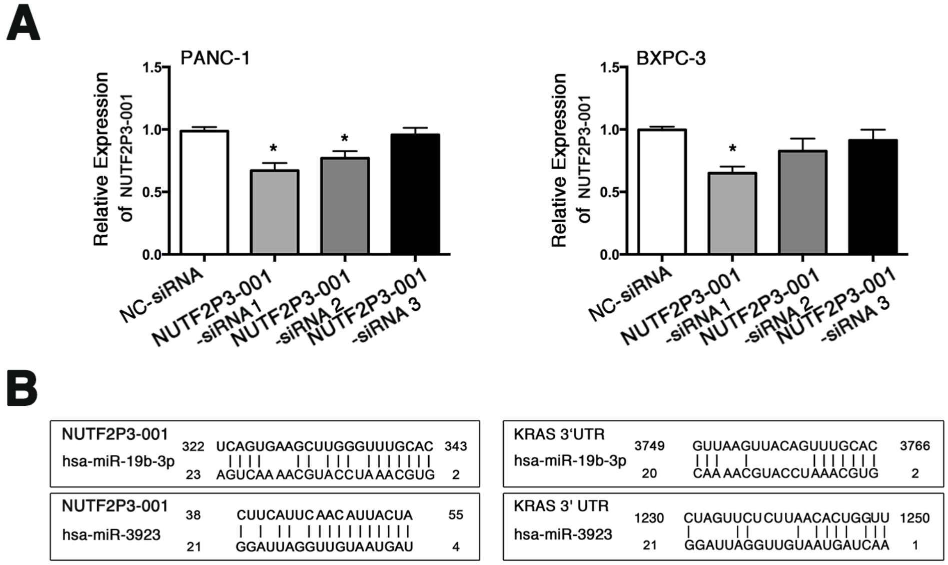

Supplementary Figure S3: The siRNA sequences selection for lncRNA-NUTF2P3-001 and potential binding sites among lncRNA-NUTF2P3-001, miRNA-3923/miR-19b-3p and KRAS mRNA. (**A**) Three NUTF2P3-001-siRNA (50nM) and corresponding negative control (NC-siRNA) were designed and the most effective one (NUTF2P3-001-siRNA 1) was chosen to fulfill the following experiment. (**B**) Potential binding sites among NUTF2P3-001, miRNA-3923/miR-19b-3p and KRAS mRNA. All data were presented as means ± SD of at least three independent experiments. The *p*-value represents the comparison between groups (*$p < 0.05$, **$p < 0.01$).

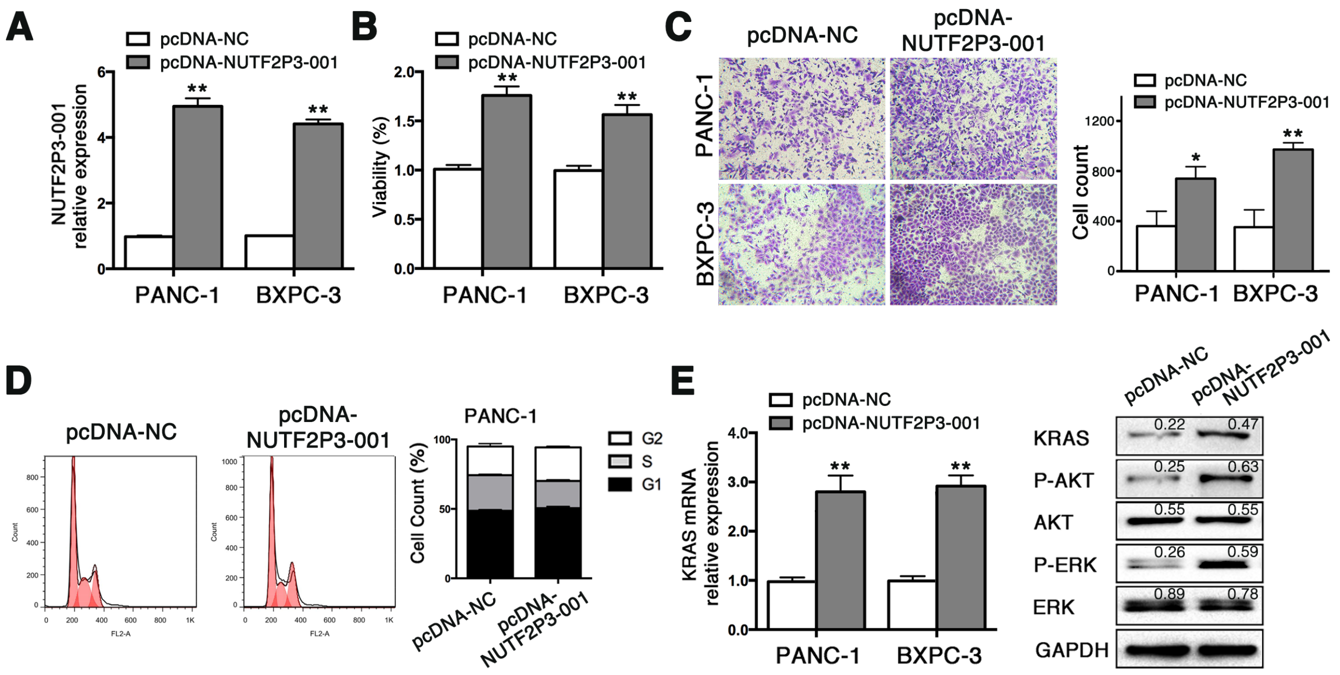

Supplementary Figure S4: Overexpressed lncRNA-NUTF2P3-001 promoted viability, proliferation and invasive ability, while induced S phase arresting in pancreatic cancer cell. (**A**) 48 h post transfection, total RNA was extracted and qRT-PCR was used to identify the efficiency of transfection with pcDNA3.1 containing NUTF2P3-001 sequence (pcDNA-NUTF2P3-001) in pancreatic cancer cell. (**B**) 72 h post transfection, MTT assays were performed and the results demonstrated that pcDNA-NUTF2P3-001 remarkably promoted viability in pancreatic cancer cell. (**C**) The capacity of invasion was identified by Transwell assay 48 h after transfection. (**D**) The cell cycle distribution was measured by flow cytometry in PANC-1 cells. The results showed that pcDNA-NUTF2P3-001 induced compromised accumulation of S-phase. (**E**) pcDNA-NUTF2P3-001 obviously upregulated KRAS mRNA expression both in PANC-1 and BXPC-3 cell lines. KRAS and its downstream proteins in PANC-1 treated with pcDNA-NUTF2P3-001 were tested by western blot in PANC-1 cells. KRAS, p-AKT/AKT and p-ERK/ERK were significantly activated. Relative intensity value is marked. All data were presented as means ± SD of at least three independent experiments. The p-value represents the comparison between groups (*$p < 0.05$, **$p < 0.01$).

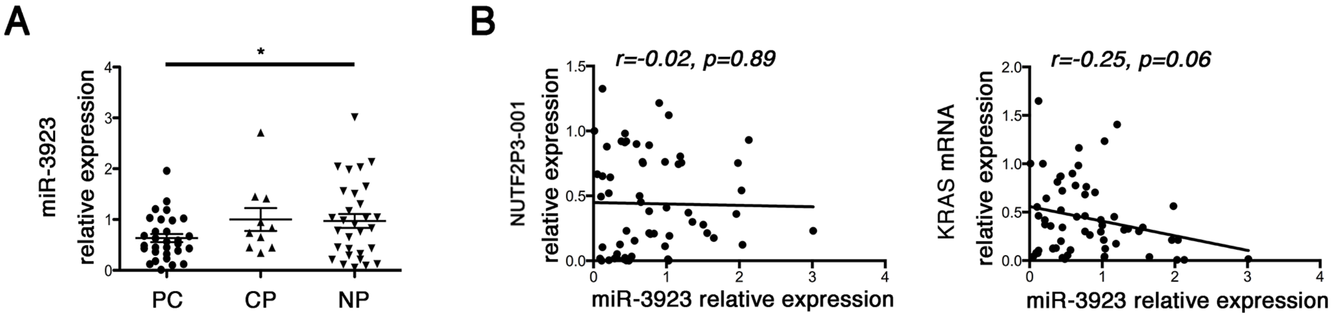

Supplementary Figure S5: The expression of miR-3923 in pancreatic tissues and correlations with lncRNA-NUTF2P3-001 or KRAS mRNA. (**A**) The expression of miR-3923 in 30 normal pancreas (NP), 10 chronic pancreatitis (CP) and 30 pancreatic cancer (PC) tissue samples was tested by qRT-PCR. Compared with normal pancreas (NP), miR-3923 was obviously decreased in pancreatic cancer (PC) specimens. (**B**) The correlations between miR-3923 and NUTF2P3-001 or KRAS expression in pancreatic tissues. The p-value represents the comparison between groups (*$p < 0.05$, **$p < 0.01$).

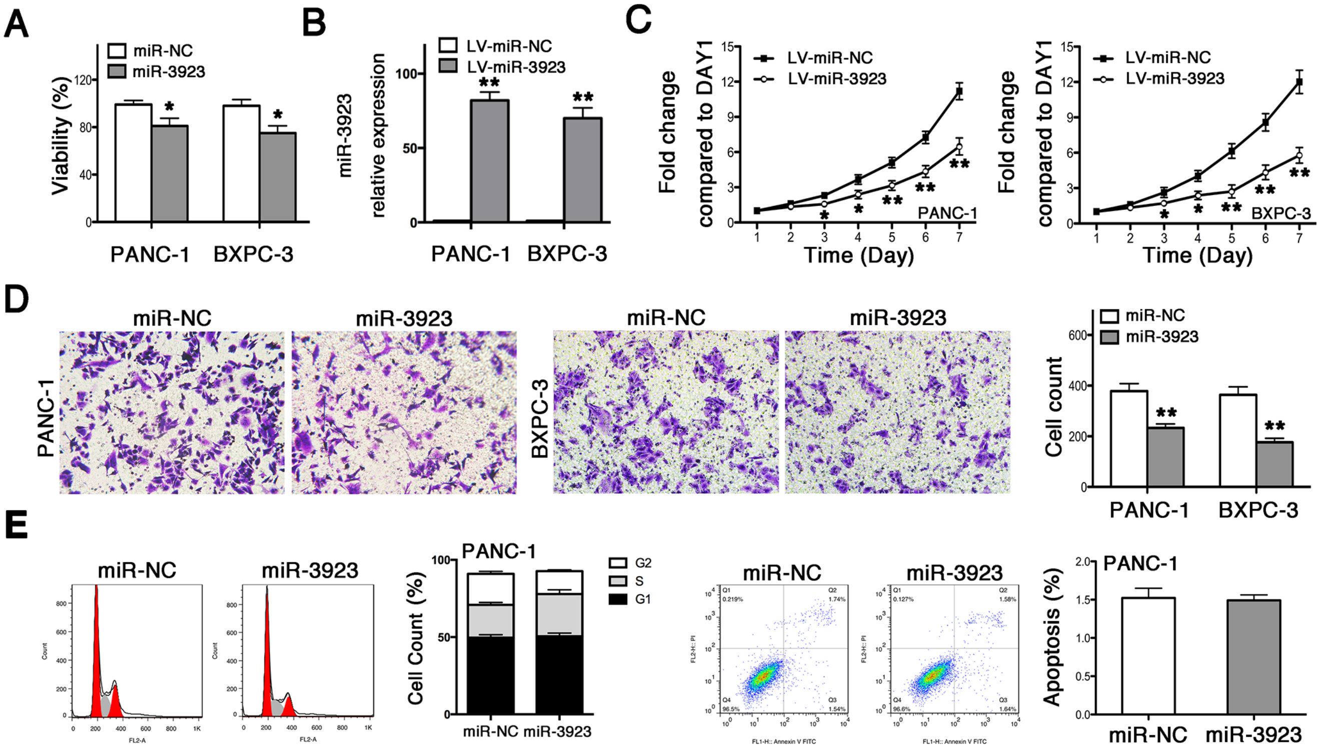

Supplementary Figure S6: Overexpression of miR-3923 induced inhibition of proliferation and invasion, as well as S phase arresting in pancreatic cancer cell. (**A**) The effect of overexpressed miR-3923 on viability of pancreatic cancer cell was examined by MTT assay. The restore of miR-3923 (50nM) could remarkably prohibit the viability of pancreatic cancer cell. (**B**) The efficiency of LV-miR-3923 transfection was measured by qRT-PCR in pancreatic cancer cell. (**C**) Pancreatic cancer cell was transfected with LV-miR-3923 and LV-miR-NC respectively by 7 days' observation. Cells in LV-miR-NC group proliferated much faster than that of LV-miR-3923 group. (**D**) After treated with miR-3923 mimics (50 nM) or miR-NC (50 nM), the invasive ability of Pancreatic cancer cell was measured by Matrigel invasion assays. The invasion capacity of pancreatic cancer cell was significantly weakened due to overexpressed miR-3923. (**E**) Pancreatic cancer cell was treated as above, and cell cycle distribution and apoptosis were measured by flow cytometry. Obvious S-phase block was detected in miR-3923 treated PANC-1 cells. All data were presented as means ± SD of at least three independent experiments. The *p*-value represents the comparison between groups (**p* < 0.05, ***p* < 0.01).

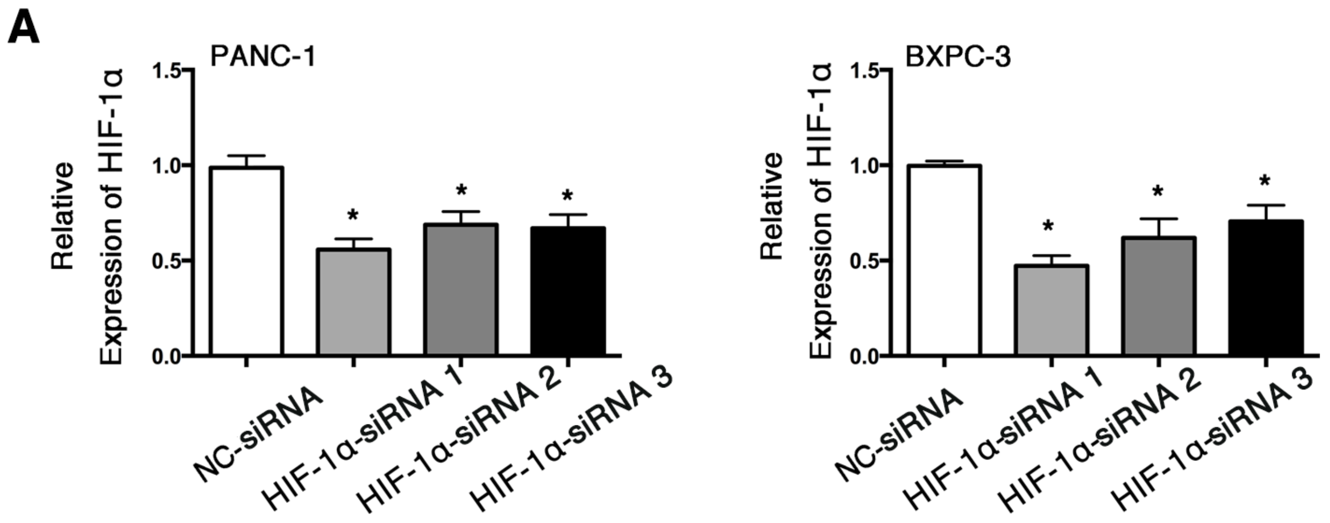

Supplementary Figure S7: SiRNA sequences selection for HIF-1α. (**A**) Three HIF-1α-siRNA and corresponding negative control (NC-siRNA) were designed and the most effective one (HIF-1α-siRNA 1, 50 nM) for both PANC-1 and BXPC-3 was chosen to fulfill the following experiment. All data were presented as means ± SD of at least three independent experiments. The *p*-value represents the comparison between groups (**p* < 0.05, ***p* < 0.01).

Supplementary Table S1: SiRNA Sequences, cloning sequence and primers for NUTF2P3-001 cloning

SiRNA Targets	Sequences
NUTF2P3-001-siRNA1 sense	5′-GAGAUAAGCUGAUUUGGAA dTdT-3′
NUTF2P3-001-siRNA1 antisense	5′-dTdT CUCUAUUCGACUAAACCUU-3′
NUTF2P3-001-siRNA2 sense	5′-CGAAUAGGUACAAUUUACA dTdT -3′
NUTF2P3-001-siRNA2 antisense	5′-dTdT GCUUAUCCAUGUUAAAUGU-3′
NUTF2P3-001-siRNA3 sense	5′-CACAGGCACAGGACAAUUA dTdT-3′
NUTF2P3-001-siRNA3 antisense	5′-dTdT GUGUCCGUGUCCUGUUAAU-3′
HIF-1α-siRNA1 sense	5′-CUGAUGACCAGCAACUUGA dTdT -3′
HIF-1α-siRNA1 antisense	5′- dTdT GACUACUGGUCGUUGAACU-3′
HIF-1α-siRNA2 sense	5′- CUGAUGACCAGCAACUUGA dTdT-3′
HIF-1α-siRNA2 antisense	5′- dTdT GACUACUGGUCGUUGAACU-3′
HIF-1α-siRNA3 sense	5′- CAAUCAAGAAGUUGCAUUA dTdT-3′
HIF-1α-siRNA3 antisense	5′- dTdT GUUAGUUCUUCAACGUAAU-3′

LncRNA-NUTF2P3-001 clone sequence in pcDNA3.1 XhoI / BamHI
5′-ATGGGAGATAAGCTGATTTGGAAGCAAACTGAATCCAGCTTCATTCAACATTACTACCAGTTATTTGATA-AAGATACAACCCGAATAGGTACAATTTACATTGACGCGTCATGCCTTAGGTGGGAAGGACAGCAATGA-CAGGGGAAAGCTGCCATTGTGGAGAAGTTGTCTAGCCTTCTGTTCCAGAAAATCCAGTACAGCATCACAG-GCACAGGACAATTAGCCCACACCAGATAGCTGCATCATCAGCATGGTTGTGGGTGAGCTTAAGGTAGAT-GAAGACCCCATCATCGGGTTCCACCAGATGTTCCTATTAAAGAATATCAGTGAAGCTTGGGTTTGCACCAAT-GACATGTTCAGGCTTGCCCTGCTCACTTCGGCTGA-3′

Primers for cloning:
Sense: 5′- ACCGCTCGAGATGGGAGATAAGCTGATTTGGAAG -3′
Antisense: 5′- CCATGGATCCTCAGCCGAAGTGAGCAGGGCAAG -3′
